# Smart Solutions: A New Direct Current Electric Machine Design to Reduce Weight and Enhance Efficiency

**DOI:** 10.1002/gch2.202500167

**Published:** 2025-10-28

**Authors:** Dmytro Ivliev, Iryna Bashynska, Volodymyr Kosenkov, Andrii Boiko, Liubov Niekrasova, Ivan Chepovskyi

**Affiliations:** ^1^ Department of Electromechanical Engineering Odesа Polytechnic National University Odesa 65058 Ukraine; ^2^ Department of Organizational Management and Social Capital AGH University of Krakow Krakow 30‐059 Poland; ^3^ Department of Physics and Electrical Engineering Khmelnytskyi National University Khmelnytskyi 29000 Ukraine; ^4^ Department of Economics Odesа Polytechnic National University Odesa 65058 Ukraine

**Keywords:** armature reaction, brush DC motor, efficiency, induction motor, smart solutions, weight

## Abstract

The main use of brush direct current (DC) motors has traditionally been in variable speed applications. Although the introduction of variable‐frequency systems shifted the market toward alternating current (AC) technologies, direct current (DC) solutions remain relevant, especially with solar power generation where DC operation can avoid inverters. However, the high cost of brush DC motors limits broader use, creating a need for designs with higher efficiency, lower cost, and simplified manufacturing. A promising solution is the direct current motor with a winding‐free rotor (DCFR). This design eliminates commutating poles and compensating windings while maintaining high overload capacity, and it doesn’t require expensive permanent magnets. Since all windings are stationary, the DCFR can operate with either a commutator or an electronic controller. Research results demonstrate clear advantages of the electronically controlled DCFR compared to a classic DC motor. With a rated power of 5.9 kilowatts and a nominal speed of 1490 revolutions per minute (rpm), the DCFR weighs only 60 kg versus 76 kg for the conventional design. Efficiency reaches 91.8%, compared to 85%. These improvements in efficiency, reduced weight, and lower material costs indicate that the DCFR design is highly suitable for both variable speed operations and solar energy applications.

## Graphical abstract

This article introduces a smart solution for the design of a brush DC motor with a winding‐free rotor (DCFR). The proposed model simplifies manufacturing, reduces material costs, and enhances efficiency. Designed for variable speed and solar power applications, the DCFR achieves 91.8% efficiency and a 21% weight reduction compared to conventional models, supporting economic and environmental sustainability.

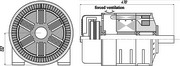

## 1 Introduction

According to an International Energy Agency (IEA) report, between 2010 and 2023 the installed capacity of solar photovoltaic (PV) systems expanded 40‐fold, while that of wind power grew six‐fold.^[^ ^1^ ^]^ The IEA forecasts that by 2035 26% of global electricity generation will be provided by solar PV systems.^[^ ^2^ ^]^ According to a study,^[^ ^3^ ^]^ if grid balancing challenges due to the intermittency of wind and solar power are addressed with enhanced storage, grid connections and demand‐response policies, solar photovoltaic (PV) systems could supply ≈56% of global electricity generation by 2050. Integrating smart loss management techniques like DNN‐based forecasting in distribution networks could further reduce system‐wide inefficiencies and promote DC adoption.^[^ ^4^ ^]^

In practice, achieving this will lead to the creation of a new cluster in the global energy system, one based primarily on direct current (DC) rather than alternating current (AC). This development necessitates a reevaluation of the role of brush DC motors in industrial production.

In 2017, Lawrence Berkeley National Laboratory released a study entitled “DC Appliances and DC Power Distribution: A Bridge to Future Net Zero Energy Homes.”^[^ ^5^ ^]^ The study points out that in the United States alone more than a million households already use solar power. In these homes energy undergoes a two‐stage conversion. First, the DC from solar PV modules is converted into AC by an inverter. Then, the AC is converted back into DC by AC/DC adapters (in computers and other electronic devices). Double energy conversion can be avoided if a house is equipped with appliances that run on DC. Such appliances are more efficient because they do not require energy to be converted twice, so switching to DC can save up to 30% of a household's total energy consumption. Instead of induction motors, these appliances use brushless direct current motors (BLDC). These reliable, compact and highly efficient motors have proven themselves both in household appliances and in the automotive industry. However, BLDC motors have drawbacks. Specifically, their control systems are complex and require specialized knowledge and have a higher initial cost.^[^ ^6^ ^]^ These factors can prevent them from being adopted, even in situations in which they would otherwise have a clear advantage over induction motors.

A striking example is the solar pump market, a young and rapidly developing sector. The global solar pump market, which was valued at approximately USD 2.77 billion in 2024, is projected to reach around USD 5.34 billion by 2034, with a compound annual growth rate (CAGR) of 6.80% between 2024 and 2034.^[^ ^7^ ^]^ As the name implies, a solar pump system is powered by a solar PV system, and it is used to supply water for irrigation or to provide drinking water. A solar pump can be equipped with either an induction motor or a BLDC motor. BLDC pumps do not require an inverter, which gives them higher efficiency compared to AC pumps and so reduces the number of solar panels needed. Nevertheless, AC pumps still account for ≈67% of sales and DC pumps account for only 33%.^[^ ^7^ ^]^ The main reason is the higher cost of BLDC motors. The combination of an AC pump plus an inverter is often cheaper. Moreover, the high cost of BLDC motors limits the range of tasks they can carry out. The power range of DC pumps is typically 1–4 kW, whereas for AC pumps it is 1–8 kW.^[^ ^7^ ^]^ Using a brush DC motor in place of a BLDC motor would only make matters worse by adding low efficiency to the already high price.

Today, at least four types of AC electrical machines are widely used in industry. They provide efficiency and flexibility in various applications. However, in the rapidly growing solar energy market, the situation is different. Only BLDC and permanent magnet DC motors (PMDC motors) can directly utilize power from solar panels. These motors occupy the niche of expensive high‐efficiency applications. Currently, there is no cost‐effective efficient DC motor in the lower‐price market sector. Consequently, a new type of DC motor with efficiency, weight and cost that match those of induction motors needs to be developed. Such a motor would more fully leverage the energy‐saving potential of solar power and increase environmental safety by reducing or even eliminating permanent magnets in its design.

According to an analysis of the 2024 BLDC market,^[^ ^6^ ^]^ 50% of all sales were of motors rated below 0.75 kW, and the rotation speeds most demanded ranged from 2000 to 10 000 rpm. A new DC motor design that performs efficiently at 600–2000 rpm speed and 1–10 kW power level would significantly expand the range of DC motor applications in both solar power and industrial production. The aim of the research presented in this article is precisely to develop such a design. Because the proposed design is new, it is logical to apply numerical and experimental methods of investigation, including modeling electromagnetic and thermal processes, and full‐scale testing of a prototype.

## 2 Literature Review, Materials, and Research Methods

### 2.1 Design and Operation Principles

The design of a direct current motor with a winding‐free rotor (DCFR) is largely similar to that of a homopolar inductor alternator (HIA). Initially, DCFRs were developed as linear direct current motors, with a magnetic circuit designed by A. Ivliev and special direct current windings designed by V. Kosenkov.^[^ ^8^ ^]^ The excitation winding and the armature winding are located on the movable part of the motor. The secondary stationary part of the motor consists of a steel strip, on which non‐wound poles are arranged in a staggered pattern. Based on this design, between 1970 and 1990 thermal cutting machines, metal laser‐cutting machines and a motor for transmitting rotation to a sealed volume through a non‐magnetic partition were manufactured, tested and delivered to customers.

**Figure 1:**
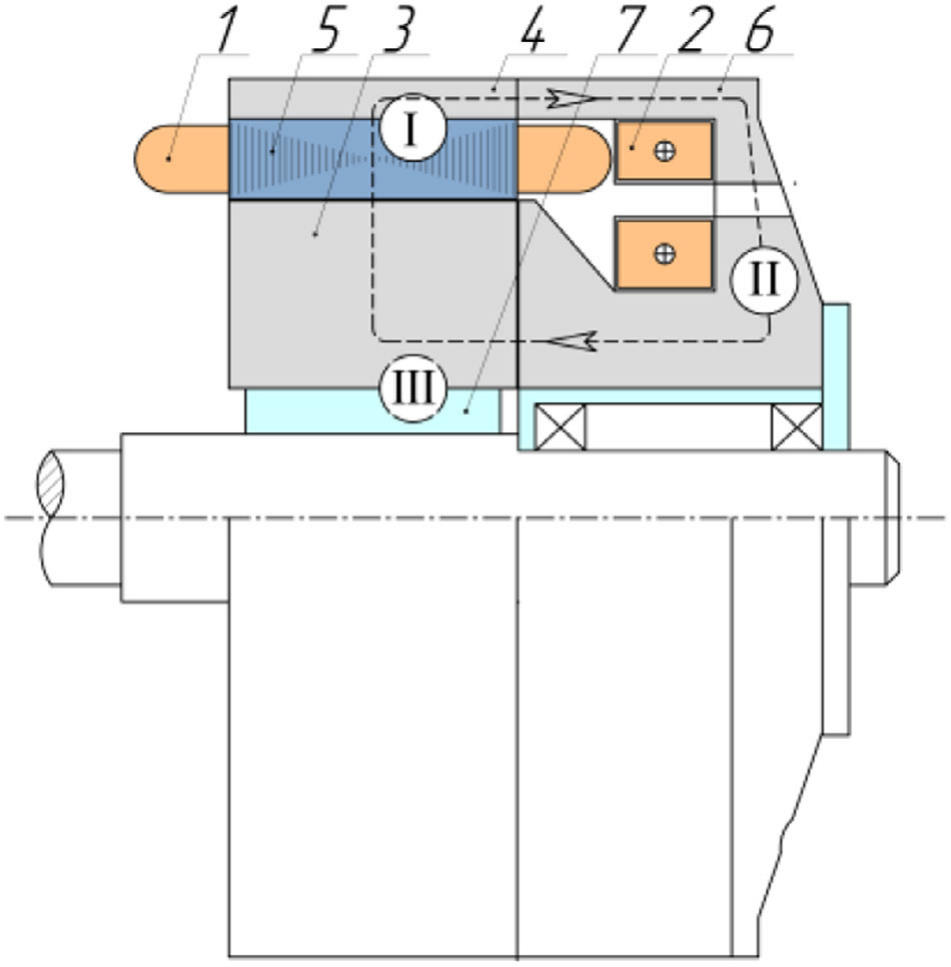
Direct current motor with a winding‐free rotor.

**Figure 2:**
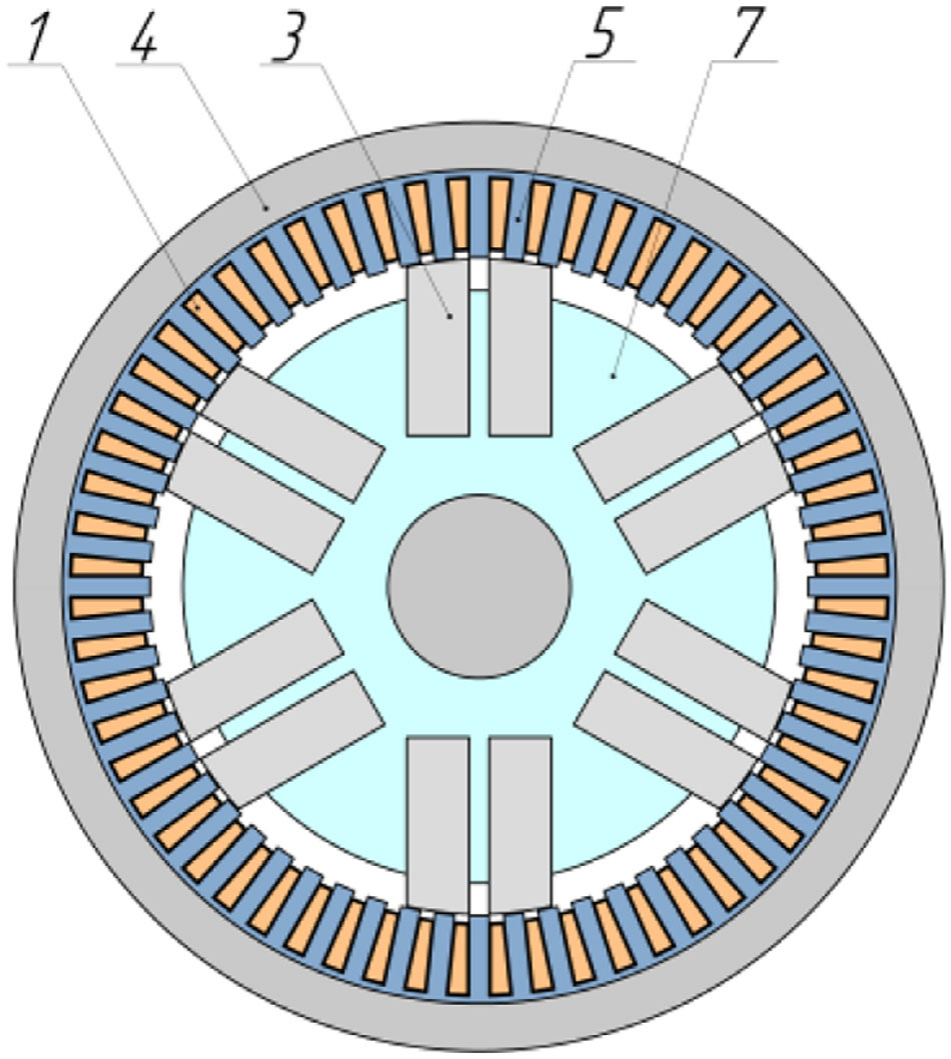
Cross‐sectional view of a DCFR motor.

As a result of a topological transformation of the secondary part of the linear motor from a strip into a cylinder, a rotary‐type DCFR design was obtained (**Figures** **1** and **2**).

In the DCFR design (Figures 1 and 2), both the armature winding (1) and the field winding (2) are stationary. The yoke (4) and flange (6) are made of solid ferromagnetic material. The poles (3) can be either solid or laminated from electrical steel sheets to reduce weight. In both cases, the poles incur no iron losses as they do not undergo magnetization reversal. Only the stator core made of electrical steel (5) experiences this. All poles (3) are fixed to the aluminum rotor hub (7) (Figure 2).

The magnetic induction in the air gap of the DCFR varies only in magnitude while its direction remains constant. Therefore, only half the section participates in energy conversion,^[^ ^9^ ^]^ which necessitates an increase in the length of the stator core and the section.

As in a HIA, energy conversion in the DCFR occurs due to the change in mutual inductance between the armature winding and field windings when the rotor poles move relative to the stator teeth.^[^ ^9^ ^]^

However, the DCFR has notable differences:

1.  the armature winding is not a three‐phase AC winding but a lap DC winding;
2.  unlike a HIA, in which end winding leakage flux does not affect the excitation winding flux, in the DCFR this influence must be accounted for.

Since the armature winding in the DCFR is stationary, the direction of current in the coils can be switched using either an inverted mechanical commutator or an electronic controller.

### 2.2 Methods to Reduce the Armature Reaction Field

**Figure 3:**
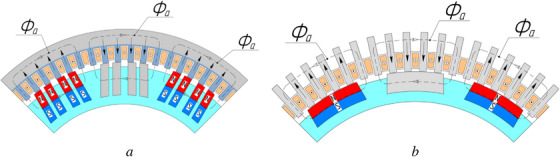
Segmentation: a) poles; b) stator yoke.

The most radical method to reduce the armature reaction in DC motors is to install compensating windings. Another significant method involves increasing the air gap, which raises the magnetic reluctance along the path of the transverse armature reaction flux. In some designs, such as DC motors with printed windings on the armature (e.g., pancake motors), increasing the air gap is the primary approach. However, this method has a major drawback: due to the 2D nature of the flux closure paths of the main magnetic flux and the armature reaction flux, increasing the gap automatically necessitates a 1.3–1.4 times increase in the magnetomotive force (MMF) of the field winding. In the DCFR, the flux closure paths are 3D, making it possible to reduce the armature reaction flux without increasing the air gap. The reduction of the armature reaction flux is achieved by segmenting either the poles (**Figure** **3**a) or the stator yoke (Figure 3b).

This design solution significantly reduces the armature reaction flux Фа by increasing the magnetic resistance of the magnetic core in the transverse direction. Therefore, the DCFR design does not require commutating poles or compensating windings to be installed, which allows the air gap to be minimized.

### 2.3 Classification of the DCFR

The DCFR design has several distinctive features:

1. An option to use either a commutator or an electronic controller
2. Segmentation of the magnetic core
3. Electromagnetic excitation (EME) or hybrid excitation (HE)

With EME, depending on the specific design, 50–66% of the active part of the stator takes part in energy conversion. If permanent magnets are set in the pole‐pitch gaps that are free of poles, then effectively the entire stator is involved in energy conversion (Figure 3). Assuming that the motor was originally designed with only electromagnetic excitation, this solution allows the power of the motor to be doubled without changing its geometry. A negative factor, however, is that the remagnetization frequency also doubles, which increases iron losses.^[^ ^10^ ^]^

Therefore, a more appropriate approach is to incorporate hybrid excitation in the DCFR design stage. This makes it possible to reduce the armature diameter, the number of poles, the remagnetization frequency and the overall mass of the motor. Such a motor will have 50% less permanent magnet mass compared to, for example, a BLDC and will still allow 50% motor speed control through field weakening.

**Figure 4:**
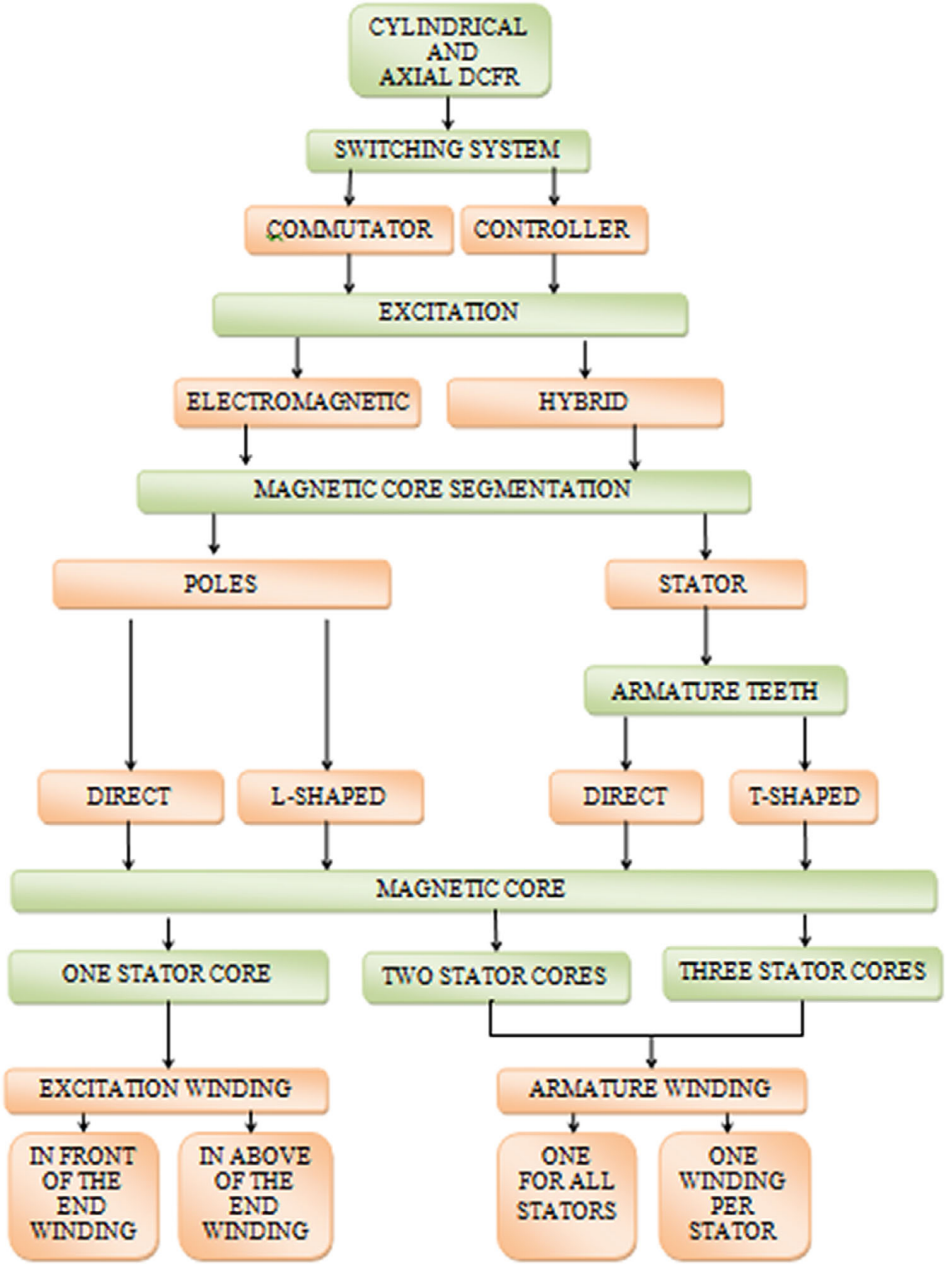
DCFR classification.

Possible designs of rotational DCFR motors are shown in the classification (**Figure** **4**). This classification reflects general approaches for both cylindrical and axial configurations.

Analyzing the classification, it can be seen that at the magnetic core level alone there are six design variants. Taking into account magnetic core segmentation increases that number to 12, and considering electromagnetic excitation or hybrid excitation raises it to 24. A review of the advantages and disadvantages of all these variants would significantly expand the scope of this article. Therefore, we plan to devote a separate article to these questions. It will include a comparative analysis of the designs and potential optimization strategies. Consequently, optimization is not discussed in this paper. A partial comparison, insofar as is possible in this article, is presented in Section 2.7.

### 2.4 Increasing Electromagnetic Loads, the Number of Poles and the Rotor Pole Embrace in the DCFR

A serially produced 2PN100 DC motor with 1 kW rated power and 1500 rpm rotational speed has Da = 9.5 cm armature diameter, Dy = 15 cm inner yoke diameter, and 2p = 2 poles.

**Figure 5:**
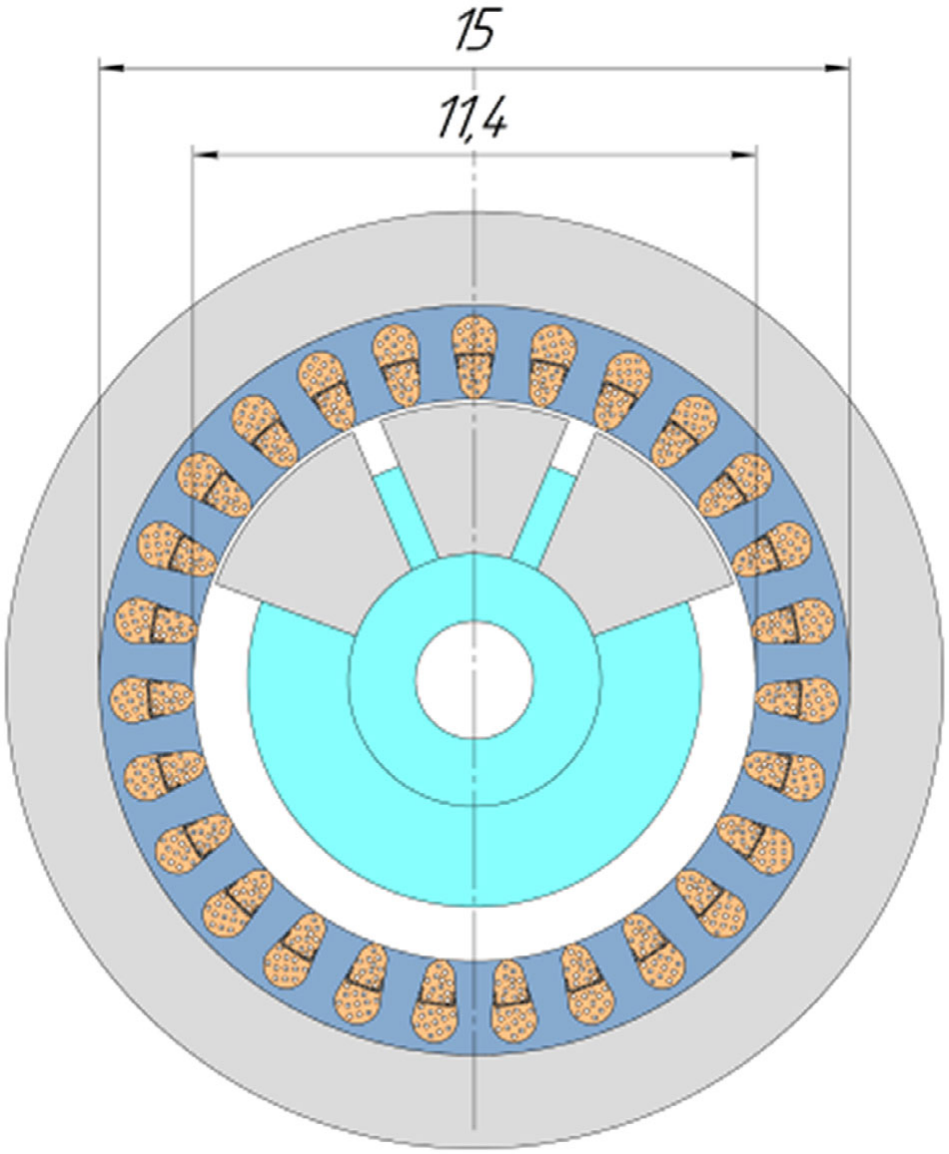
DCFR with Da = 11.4 cm armature diameter.

Unlike the classic DC motor, the DCFR has a single axial field winding shared by all poles (see Figure 1) and no commutating poles. This design allows the armature diameter to be increased from 9.5 to 11.4 cm without changing the inner diameter of the yoke (see **Figure** **5**).

Increasing the armature diameter reduces the armature stack length ℓa. In addition, the absence of commutating poles allows the rotor pole embrace αδ (pole arc to pole pitch ratio) to increase, further reducing the ℓa. This approach compensates for the need to increase the active length of the coil (see Section 2.1). Tsyplenkov et al. (2020)^[^ ^11^ ^]^ provide the following electromagnetic load values based on the design and operation of brush DC motors:

1.  Da = 95 mm, A = 110 A cm^−1^, Bδ = 0.5T
2.  Da = 114 mm, A = 130 A cm^−1^, Bδ = 0.55T

It is important to note that these values assume an absence of compensating windings in motors in this power range. However, the presence of magnetic core segmentation in the DCFR (in which one pole is divided in three segments, see Figure 5) allows the A and Bδ values to increase by at least 5–10%.

Increasing the armature diameter while maintaining the same number of poles leads to inefficient copper use due to the longer end winding coils and higher copper losses. To address this, the DCFR design (see Figure 5) recommends increasing the number of poles from 2p = 2 to 2p = 4.

The elimination of salient poles is not an entirely novel approach, as a similar concept is applied in permanent magnet DC motors (PMDC) and interior permanent magnet synchronous motors (IPMSM). Using permanent magnets significantly improves efficiency while reducing size and weight. However, IPMSMs are burdened by complex and expensive control systems, which cannot regulate or disable the magnetic flux of permanent magnets during emergency operation.^[^ ^12^ ^]^ In addition, the cost of permanent magnets is ≈$126.2 kg^−1^,^[^ ^13^ ^]^ which is significantly higher than the cost of copper, which is around €8.23 kg^−1^ ($9.19 kg^−1^).^[^ ^14^ ^]^ Unlike IPMSMs, the DCFR uses electromagnetic excitation. While its efficiency is slightly lower than that of IPMSMs, it has a significant advantage in terms of the cost of the active materials.^[^ ^9^ ^]^

### 2.5 Commutation in the DCFR

In brush DC motors, satisfactory commutation conditions are typically achieved by employing commutating poles. However, commutating poles may be omitted if the following condition is met:^[^ ^11^, ^15^ ^]^

(1) $$e_{r}+e_{q}\leq 2,5÷3,5\;V$$

where *e_r_* is the reactive electromotive force (EMF) in the commutated coil of a DC motor and *e_q_* is the electromotive force from the armature reaction field in the commutated coil of a DC motor. The value of *e_r_* in the DCFR is determined similarly to that in a brush DC motor using the well‐known Pichelmayer formula:^[^ ^11^, ^15^ ^]^

(2) $$e_{r}=2\cdot w_{a}\cdot \ell_{a}\cdot A\cdot \upsilon_{a}\cdot \xi \cdot 10^{-2}V$$

where *w_a_* is the number of turns in the armature commutated coil; *ℓ_a_* is the armature stack length in cms.; *А* is the linear current density, A cm^−1^; *Ʋ_a_* is the armature rotation speed, m/s; and *ξ* is a coefficient proportional to the equivalent magnetic conductivity of the slot, the tops of the teeth and the end parts of the winding.

The electromotive force from the armature reaction field, *e_r_*, in the DCFR is also determined similarly to that in a brush DC motor:^[^ ^15^ ^]^

(3) $$e_{r}=2\cdot w_{a}\cdot \ell_{a}\cdot B_{q}\cdot \upsilon_{a}\cdot 10^{-2}V$$

where *В_q_* is the flux density in the commutation zone from the action of the armature reaction, Т.

**Figure 6:**
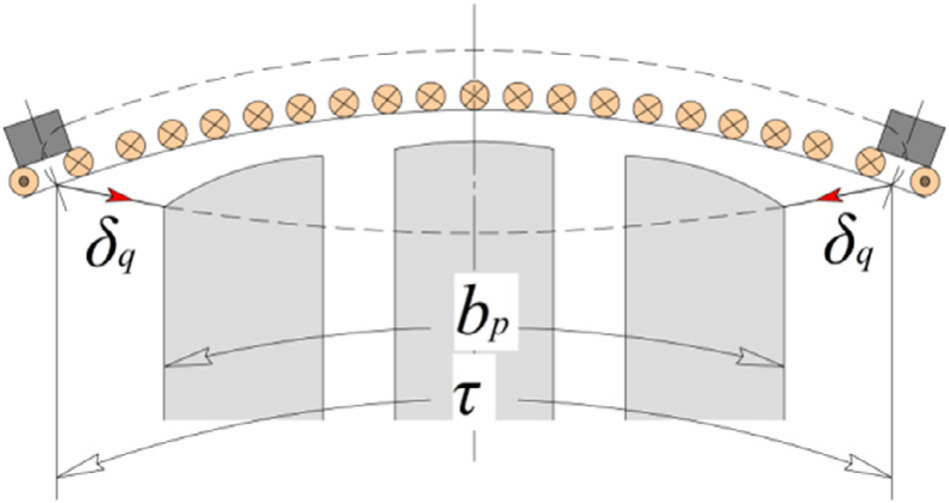
A segmented pole DCFR.

In the DCFR, due to the segmented poles, the value of *В_q_* (3) differs from that in a brush DC motor. **Figure** **6** shows a segmented pole DCFR.

The length of the magnetic line of force in the air gap portion of the circuit between the pole edge and the brush is calculated as:^[^ ^11^, ^15^ ^]^

(4) $$\delta_{q}\approx \frac{1}{2}\left(\tau -b_{p}\right)\mathrm{cm}$$

where *τ* is the pole pitch in cms and *b_p_* is the pole arc in cms.

The total length of all the air gaps is given by *k·δ_q_*, where *k* is a weighting factor, which can take both integer and fractional values. For the closed‐loop magnetic induction line shown as a dashed line in Figure 6, we have:

(5) $$\oint Hd\ell \approx \frac{1}{\mu_{0}}\cdot B_{q}\cdot k\cdot \delta_{q}=A\cdot \tau$$

where *µ_0_* is the vacuum magnetic permeability. We neglect the drop in magnetic potential in the steel sections of the circuit. From Equation (5), *В_q_* is determined as follows

(6) $$B_{q}=\frac{A\cdot \tau }{0,8\cdot 10^{6}\cdot 0,5\cdot k\cdot \left(\tau -b_{p}\right)}$$

Given that *b_p_* = α_δ_ · τ and *A* is in A cm^−1^, the equation transforms into:

(7) $$B_{q}\approx 2,5\cdot 10^{-4}\frac{A}{k\cdot \left(1-\alpha_{\delta }\right)}$$

### 2.6 Design Process of a DCFR

The design process of a DCFR is described in Ivliev (2019)^[^ ^10^ ^]^ and it largely coincides with the standard design process for DC motors. The main distinction is the need to account for the coefficient of utilization *К_u_*, the ratio of the total active length of a section to the length of that section covered by poles. Depending on the DCFR motor design, *К_u_* may take values of 0.5 or 0.66, while for hybrid excitation *К_u_* = 1.

This coefficient is incorporated in the equations for the cylindrical DCFR armature diameter *D_a_* ^[^ ^10^ ^]^ and the machine‐related constant *K_e_*.^[^ ^10^ ^]^

Armature diameter:

(8) $$D_{a}=\sqrt[{3}]{\frac{P_{a}}{1,57\cdot \alpha_{\delta }\cdot B_{\delta }\cdot A\cdot K_{u}\cdot \omega }}$$

where *D_a_* is the armature diameter, m; *P_a_* is the electric power, W; *α_δ_* is the pole arc to pole pitch ratio; *В_δ_* is the flux density in the air gap, T; А is the linear current density, A m^−1^; *K_u_* is the coefficient of utilization; and ω is the angular frequency, rad s^−1^.

Armature voltage:^[^ ^16^ ^]^

(9) $$E_{a}=K_{e}\cdot \Phi_{p}\cdot \omega$$

where *E_a_* is the armature voltage, V; *K_e_* is a machine‐related constant; and *Φ_p_* is the flux per pole, Wb.

Machine‐related constant:

(10) $$K_{e}=\frac{p\cdot N\cdot K_{u}}{2a\cdot \pi }$$

where *p* is the number of pole pairs; *N* is the number of armature conductors; *K_u_* is the coefficient of utilization; and *2a* is the number of parallel current paths in the armature winding.

The calculations of armature slots, the magnetic core, the field winding, the losses and efficiency, the machine characteristics and the thermal value remain unchanged from those in a standard DC design.

Based on data obtained from studying the stationary thermal field of the DCFR, the following values of linear current density *A* and armature‐winding current density *J* are recommended for different cooling types:

1. naturally ventilated (no fan) *А* = 200–250 A cm^−1^, *J* = 4–5 A mm^−2^;
2. own fan ventilated *А* = 300–450 A cm^−1^, *J* = 5–6 A mm^−2^.

Using this methodology, an experimental DCFR generator was designed with *P_n_* = 1 kW, *U_n_* = 289 V and *n_n_* = 600 rpm. The results of testing it confirmed the accuracy of the approach developed, with the difference between the calculated and actual parameters not exceeding 10%.

### 2.7 Comparison of the DCFR with Other Types of Electric Motors

Based on a comparative analysis of well‐known varieties of direct‐ and alternating‐current motors presented in refs. [17, 18, 19], conclusions can be drawn regarding the advantages and disadvantages of the classic brush DC motor. Overall these are as follows:

1. The brush DC motor provides the simplest control.
2. For proper operation of the brush DC motor, commutating poles and compensating windings are necessary, leading to increased mass and cost, and lower efficiency.
3. The use of a commutator reduces reliability and increases maintenance costs.
4. Most losses in a DC motor occur in the rotor, which complicates heat dissipation.

The general conclusion is that the disadvantages of the brush DC motor outweigh its advantages.

While maintaining the simplest form of control the DCFR is free of the aforementioned drawbacks. Its design does not include commutating poles or compensating windings, and all the windings are located in the stator. This improves heat dissipation and makes it possible to increase the electromagnetic load.

A convenient way to make a comparative assessment of the DCFR is to use data from a table in ref. [17]. In this publication, four types of motors are examined: a direct current motor (DC), an induction motor (IM), a permanent magnet excited synchronous motor (PMSM) and a switched reluctance motor (SRM), all of which have the same values, P = 30 kW, n = 3000 rpm and U = 400 V. The table is shown below (**Table** **1**). To provide a more complete picture, it has been expanded to include a synchronous reluctance motor (SyncRM) and a brushless direct current motor (BLDC) from ref. [20]. In addition, six DCFR calculation variants have been added to the table.

1. Option No. 1 is the baseline, corresponding to a classic brush DC motor with electromagnetic excitation and a commutator, as presented in ref. [17]
2. Options No. 2 and No. 3 are two approaches to designing the DCFR.
3. Option No. 4 is a commutator and hybrid excitation version of Variant No. 3.
4. Options No. 5 and 6 respectively represent an electronic‐controller DCFR with electromagnetic excitation and hybrid excitation.

**Table 1:** A comparative assessment of the DCFR.

	DC	IM	PMSM	SRM	DCFR					
					Commutator				Electronic controller	
					eme	eme	eme	he	eme	he
Option					№1	№2	№3	№4	№5	№6
Number of pole pairs *p*	5	2	6	12/8	6	6	6	3	6	3
Maximum efficiency *η* [%]	84	89	97	88	84.7	87.9	86.6	89.3	89.5	91.6
Rotor diameter *D_r_* [mm]	239.5	162	136.8	159	240	350	240	160	240	160
Active length *li* [mm]	69.8	127	140.8	159	134	60	100	110	80	90
Outer diameter *D_a_* [mm]	430	258	196.3	269	334	428	327	246	326	246
Inner diameter of the pole D_р_ [mm]	–	–	–	–	84	276	76	58	136	66
Length with end windings *l_a_* [mm]	132	232	161.5	207	204	157	170	207	150	187
Volume *V_a_* [dm^3^]	19.2	12.1	4.9	11.8	16.7	13.2	13.4	9.3	10.3	8.2
Power density [kW dm^−3^]	1.6	2.5	6.1	2.6	1.8	2.3	2.2	3.2	2.9	3.7
Moment of inertia J [kg m^2^]	–	–	–	–	0.12	0.12	0.08	0.03	0.07	0.03
Power density ^[^ ^20^ ^]^						Commutation condition e_r_ + e_q_ (V)				
Synchronous reluctance motor (SyncRM) −2 kW dm^−3^										
brushless direct current motor (BLDC) −3 kW dm^−3^					2.3	2.49	2.65	2.21	–	–

All the DCFR designs in these variants employ separate excitation, segmented poles and the layout shown in Figures 1 and 2. In the efficiency calculation, losses in the field winding are not taken into account. Because the air gap between the inner radius of the pole and the shaft radius in the DCFR can reach 100 mm, the active volume is determined by the outer diameter *D_а_*, the inner radius of the pole *D_ip_* and the length with end windings *l_a_*.

Ref. [17] indicates that the DC calculation was performed according to ref. [15] for *D_r_* = 239.5 mm. For that diameter, ref. [15] gives the following electromagnetic load values: *A* = 350A cm^−1^, *B_δ_* = 0,72T. These same values were used to calculate DCFR No. 1. The result of this calculation showed that with the rotor diameter *D_r_* unchanged the active length *li* is almost twice that of its counterpart, which confirms the conclusions drawn in Section 2.1 for a DCFR with a coefficient of utilization *К_u_* = 0.5. Nevertheless—but only by a small margin—the efficiency and power density values for No. 1 are still better than those of its counterpart. Further calculations were carried out for two scenarios:

1. No. 2: an increased rotor diameter *D_r_* with the base values *A, B_δ_* and the outer diameter *D_a_* unchanged.
2. No. 3: rotor diameter *D_r_* maintained but A increased by 20% to 450 A cm^−1^, which according to ref. [10] is acceptable for a DCFR with cooling method IC411.

In terms of all the indicators except the moment of inertia, No. 2 turned out to be superior, while its moment of inertia is inferior to that of No. 3. Variant No. 4 shows that with hybrid excitation it is better to reduce *D_r_* and increase *A* to 450 A cm^−1^. In this case, the armature winding commutation condition is better, the mass is lower and the efficiency is higher. For all six variants, and for the counterpart *B_δ_* = 0.72 T. Variants No. 5–6 were calculated for electronic‐controller versions with A = 450 A cm^−1^.

The comparison of Variants No. 1–6 is far from exhaustive and requires further clarification, which, as was noted above, will be presented in a separate article. Nevertheless, some preliminary conclusions can already be drawn. The commutator design of Variant No. 4 is superior in terms of both efficiency and power density. It outperforms the IM, the SRM, the SyncRM and the BLDC. However, Variant No. 2 may be cheaper than Variant No. 4 because it does not use permanent magnets. The electronic‐controller design of Variant No. 6 is the best. Its parameters are close to those of the PMSM.

## 3 Research Results

### 3.1 An Experimental Study of the DCFR

DCFR studies were conducted on two experimental prototypes: axial and cylindrical ones. The axial DCFR was designed to operate as an engine, while the cylindrical one was designed to operate as a generator. The results of the engine study are discussed in ref. [9]. Therefore, this article focuses solely on the results of the cylindrical DCFR generator study.

#### 3.1.1 The DCFR Electronic Controller

**Figure 7:**
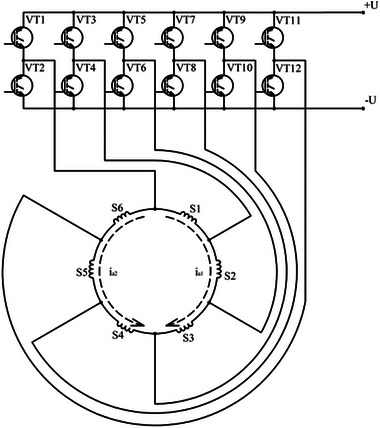
Armature winding diagram.

To test the feasibility of electronic control of the DCFR, a common electronic controller was developed for both prototypes. This operates by sequentially switching the armature winding coils (**Figure** **7**). For convenience, the operating principle of the controller is described for the engine mode.

The scheme works as follows. If the position of the poles is such that the rotor position sensor (RPS) opens the VT1 and VT8 keys, then the current passes along the following path: from the positive terminal of a power supply to the VT1 key, to sections S1–S3 (the first parallel branch), to the parallel sections S6–S4 (the second parallel branch), to the VT8 key, to the negative terminal of the power supply. In this case, the rotor turns and the RPS closes keys VT1 and VT8 and turns on keys VT3 and VT10 (or VT11 and VT6 when moving the rotor in the other direction). When keys VT3 and VT10 are turned on, the direction of the current in sections S1 and S4 is reversed. With further rotation of the rotor, the signals from the RPS enable the next pairs of transistor keys and when moving the engine at 2τ a full cycle of switching electronic keys occurs. In generator mode, the reverse conversion of energy is carried out based on the same principle.

**Figure 8:**
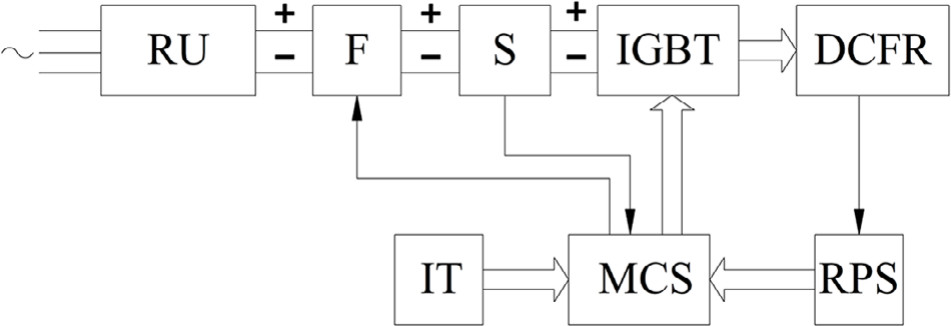
Block diagram of the DCFR electronic controller. RU = Rectifier Unit; F = Capacitor filter with braking resistor; S = current and voltage sensors; IGBT = IGBT Power Modules; IT = Input Terminal; MCS = microprocessor control system; RPS = rotor position sensor.

A block diagram of the DCFR electronic controller is shown in **Figure** **8**.

The electronic controller consists of two main components: the power stage and the control system. The power stage contains 12 power semiconductor switches (PSSs). For PSSs, two Mitsubishi PM100CLA060 IGBT Intelligent Power Modules (IPMs) are used, each of which combines six IGBTs and gate drivers.

The distinctive feature of the circuit presented is the large number of power switches, 12 instead of 6 as in a standard VFD (Variable Frequency Drive). In control systems for asynchronous motors, such as dual inverters and split‐phase inverters, 12 power switches are also used.

#### 3.1.2 Results of Testing the Experimental DCFR Sample

**Figure 9:**
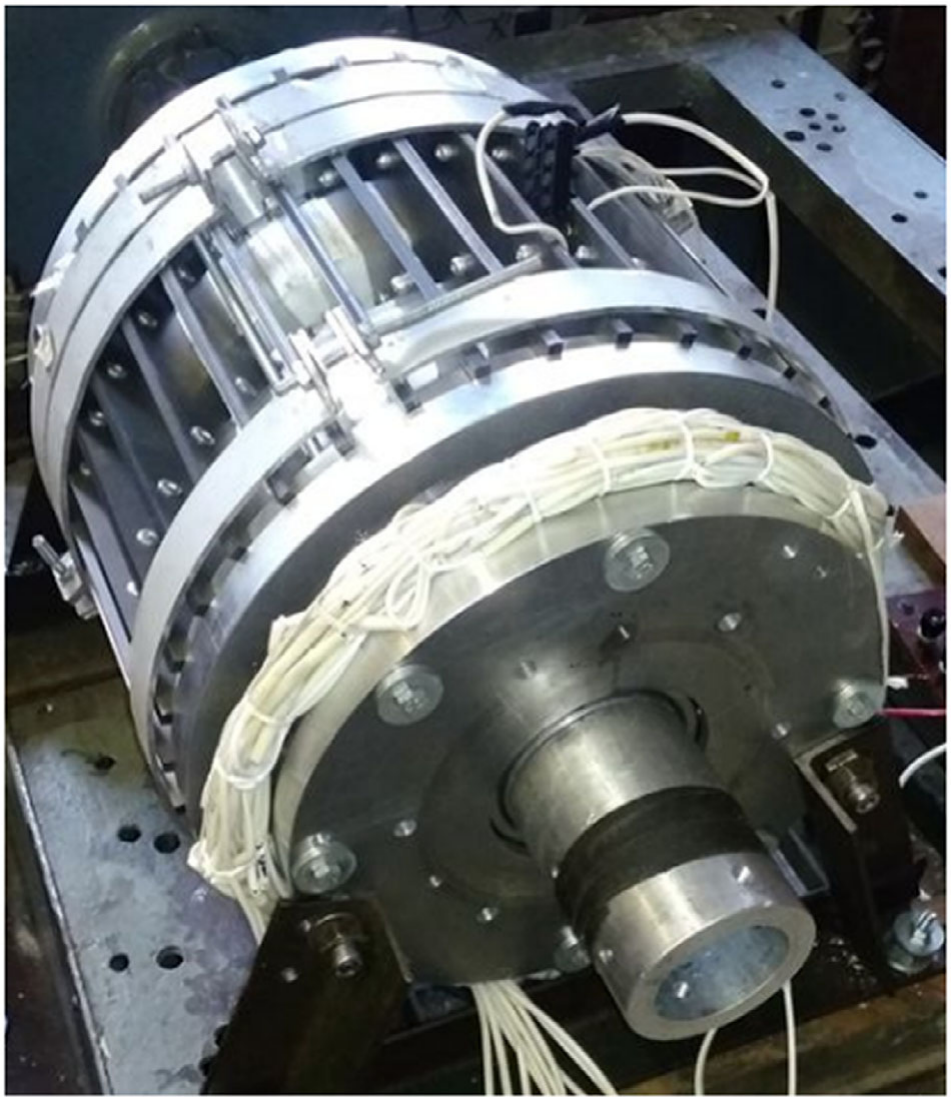
DCFR generator.

To verify the operability of the new design and the method to reduce the armature reaction field (see Section 2.2), an experimental DCFR generator was manufactured^[^ ^10^ ^]^ for use as part of a wind turbine with values Pn = 1 kW, Un = 289 V, nn = 600 rpm (**Figure** **9**). According to the classification in Figure 4, this design is a cylindrical type with electromagnetic excitation and a segmented stator, the magnetic core of which consists of three stator cores with two field windings, direct teeth and one common armature winding for all stators with utilization coefficient Кu = 0.66.

**Figure 10:**
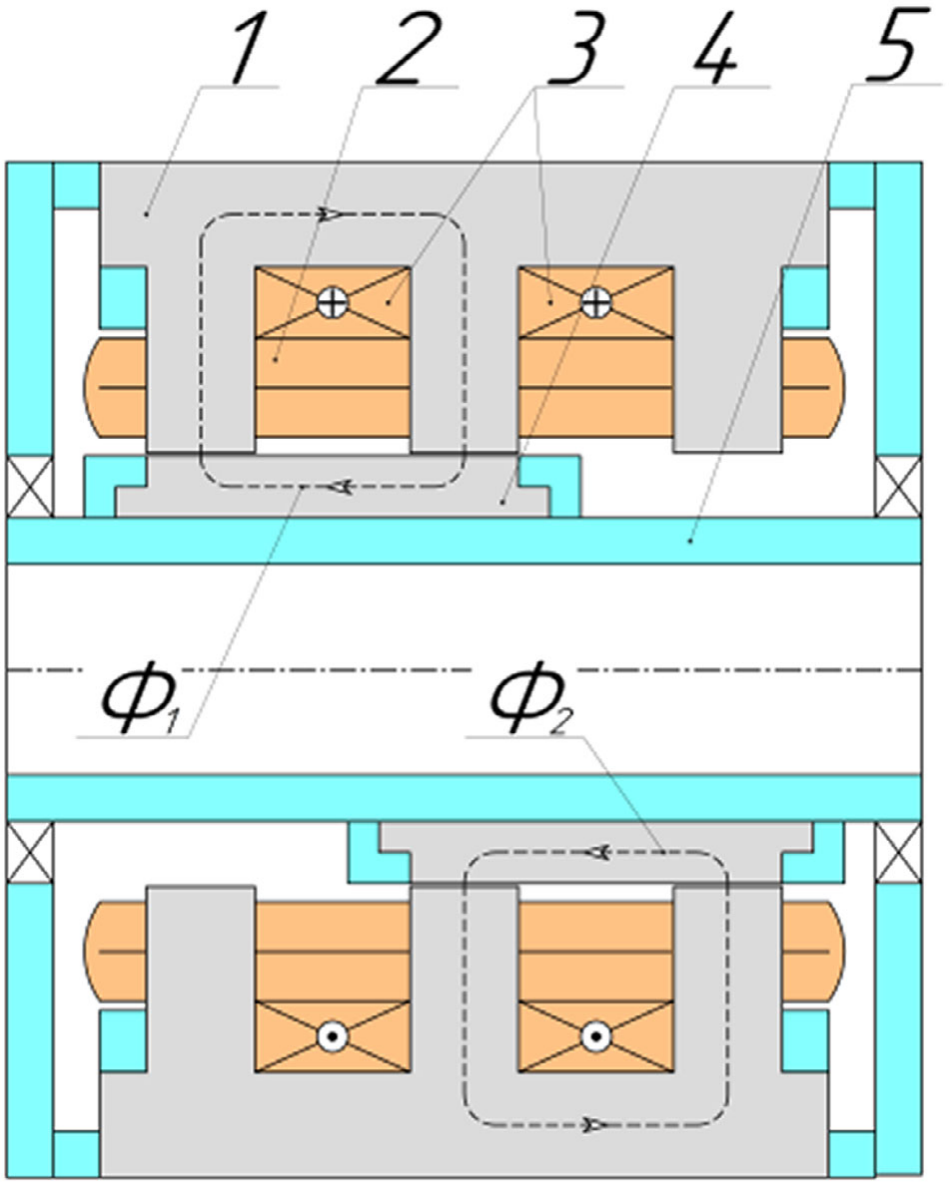
Side view of the DCFR generator.

**Figure 11:**
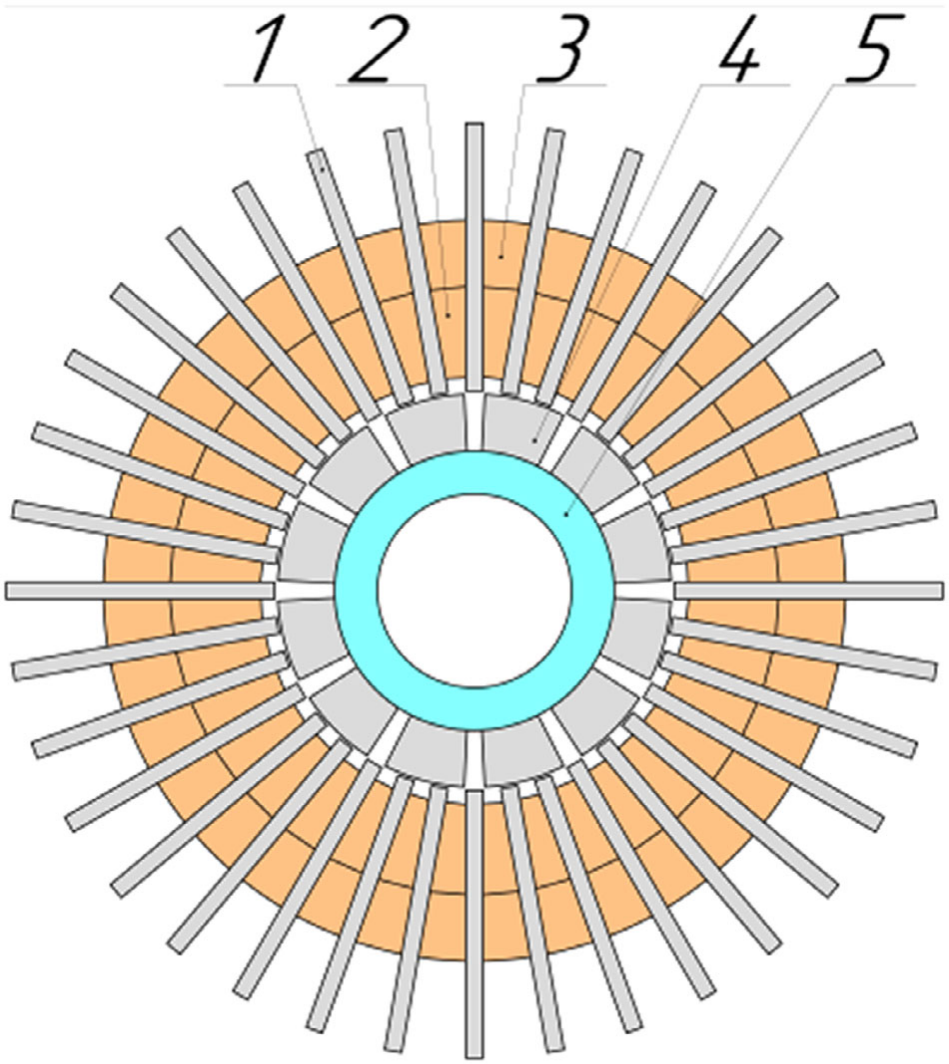
Front view of the DCFR generator.

The DCFR stator (**Figures** **10** and **11**) lacks a common yoke and consists of a series of magnetically unconnected M‐shaped teeth made of electrical steel 1), between which the sections of the armature winding 2) are placed. In addition, the stator contains two excitation windings 3), which generate two counter‐parallel magnetic fluxes. The rotor comprises twelve steel winding‐less poles 4) arranged in a staggered pattern and fixed on the aluminum shaft 5).

**Figure 12:**
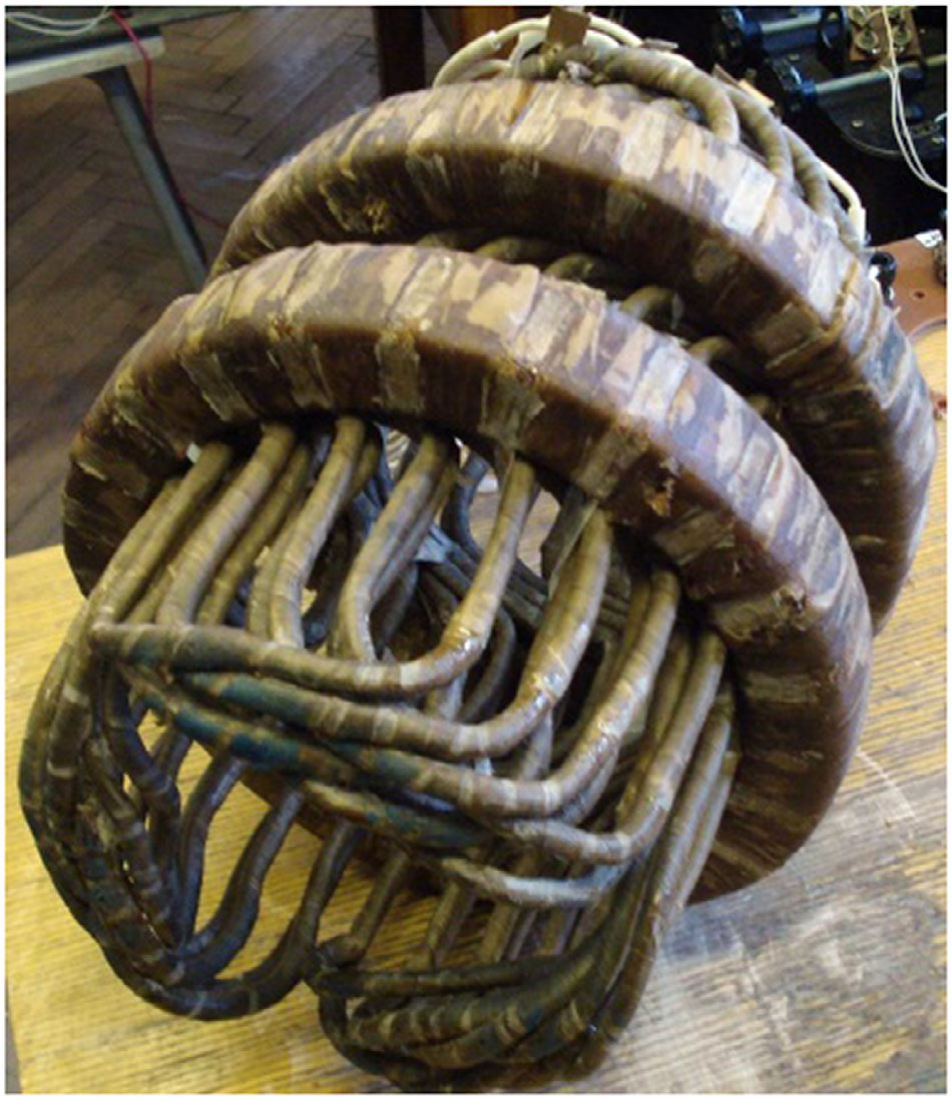
Armature winding and excitation windings after impregnation.

**Figure 13:**
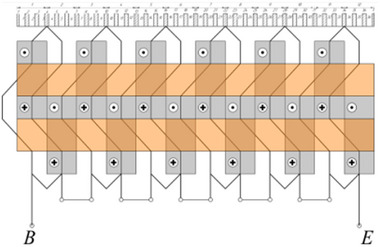
One poly‐coil DCFR.

The installation of the armature coils is carried out with a partial shift equal to the pole pitch τ (**Figures** **12** and **13**). In this case, the direction of the magnetic flux through the coil remains unchanged, which ensures that the electromagnetic torque retains its sign (Figure 13).

Most operations involved in assembling the DCFR stator can be easily automated. The exception is the installation of the armature coils, which requires preliminary bending of wound coils (Figure 13). However, this step can also be automated if the assembly sequence of the stator is changed as follows. First the coils are installed and then the M‐shaped teeth are inserted. The coils are assembled on a special wooden form and then impregnated with insulating varnish (Figure 12). After the varnish has hardened, the wooden form is removed and replaced with the M‐shaped teeth. The DCFR armature winding consists of six poly‐coils, one of which is shown in Figure 13.

The prototype was designed and manufactured based on the following conditions:

1. do not use permanent magnets;
2. minimize the weight of the DCFR;
3. use natural cooling;
4. use the electronic controller only for laboratory research
5. in the finished product, use the only rectifier circuit.

The use of natural cooling imposed limitations on the values of electromagnetic loads: A = 249 A cm^−1^ and Вδ = 0.44T, which correspond to the design recommendations for low‐speed generators in wind power installations.

Based on preliminary calculations, the armature diameter was set at Da = 14 cm.

Within the stated constraints, weight reduction was achieved by implementing a DCFR design with the maximum possible utilization factor, Кu = 0.66, which increased the number of poles to 2р = 12, and raised the pole overlap ratio to αδ = 0.8.

When using a rectifier circuit in the DCFR, it is necessary to account for the influence of the armature winding inductance on generator operation. This in turn leads to a change in the connection scheme of the enlarged sections and addition of capacitors to the circuit to create current resonance. In this case, the DCFR shifts from the class of direct current motors to that of alternating current electric motors. This transformation should not be surprising. For instance, some authors classify BLDC motors as DC motors while others consider them synchronous AC motors.

DCFR testing was carried out in three stages:

1. with one enlarged section (and all the others disconnected), to obtain data excluding the influence of inductance;
2. with an electronic controller;
3. with a rectifier circuit.

Due to the large volume of test data, this article presents only the results from the first stage, data on losses and efficiency, and thermal testing from the third stage.

In the first stage, each of the six poly‐coils was connected separately to an electrical load, while the other poly‐coils were disconnected. All the tests were performed with constant values of *n* = 600 rpm and *I_в_* = 0.65А. The measurements showed practically identical results for a single poly‐coil: *U* = 100 V, *I* = 1,6 А, *P* = 160 W, with the average deviation not exceeding 5%.

**Figure 14:**
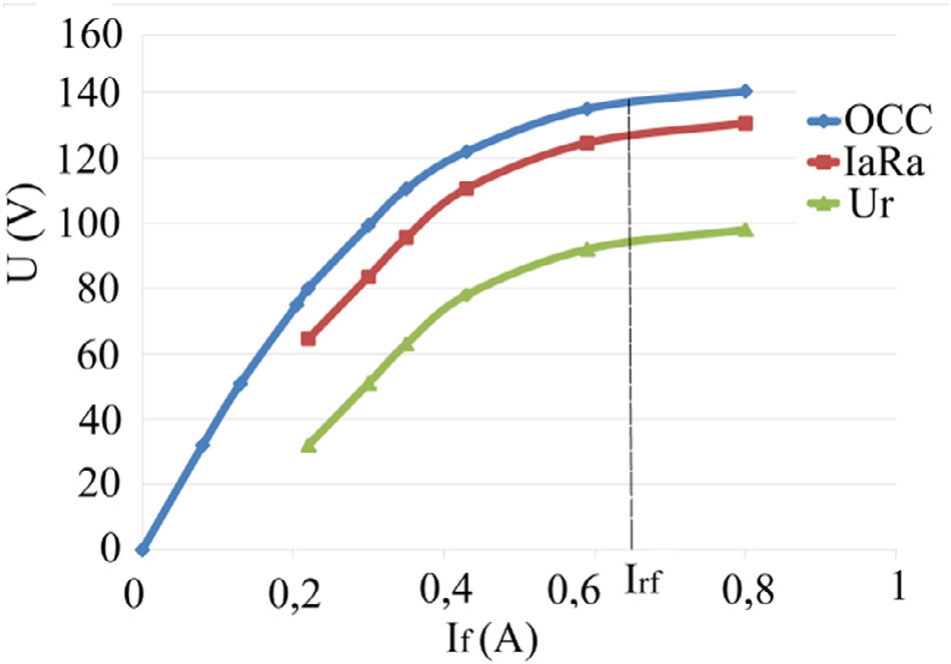
OCC and load characteristic, one poly‐coil.

**Figure 15:**
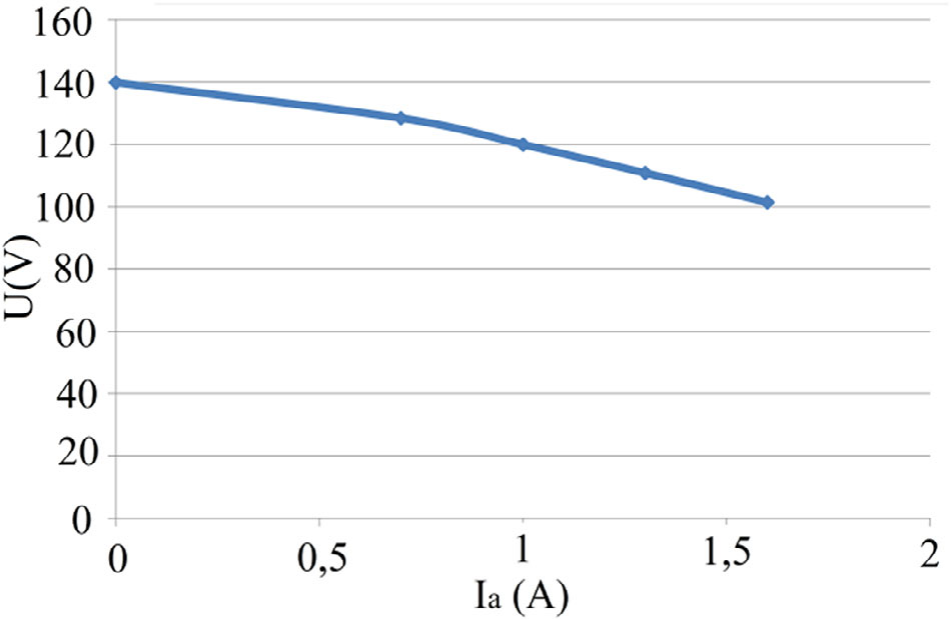
External characteristic, one poly‐coil.

**Figure** **14** shows the open‐circuit characteristic (OCC) and the load characteristic, and **Figure** **15** shows the external characteristic.

Analysis of the experimental characteristics obtained shows that the armature reaction in the generator prototype is significantly weakened. As is well known, the shape of the generator's external characteristic is influenced by two factors: the voltage drop due to armature reaction and the I_a_R_a_ voltage drop. In the absence of the first factor, this dependency becomes linear and depends only on the load current.

Based on the results of the first testing stage, the power output of a single enlarged section was 160 W. Accordingly, the total power of the DCFR with six enlarged sections amounts to 160 × 6 = 960 W. During the second testing stage with the electronic controller, this result was experimentally confirmed. The armature winding sections were connected using the electronic controller scheme (Figure 7).

**Figure 16:**
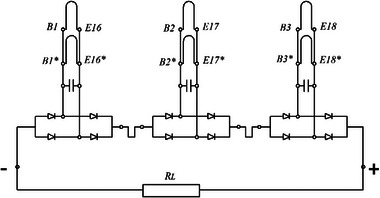
Connections of the sections to the load with a capacitor.

In the third testing stage, with the entire armature winding connected according to the scheme shown in **Figure** **16**, the results presented in **Table** **2** were obtained. The enlarged sections were grouped in three sets. Each group consisted of two enlarged sections connected in parallel and placed in the same slots. A capacitor was connected in parallel with the sections to create current resonance in the circuit (Figure 16).

**Table 2:** Experimentally obtained data.

Parameter	Unit	Experiment
Rated power *Р*	W	954
Rated voltage *U*	V	289
Rated speed *n*	rpm	600
Type excitation		separate
Frame size *h*	mm	160
Armature current *I_a_*	А	3,3
Armature resistance R_a Т = 75С_	Ω	29,3
Field current *I_f_*	А	0,6
Field resistance *R_f_*_Т = 75С_	Ω	289
Excitation voltage *Ue*	V	173
Copper losses in armature winding *Р_a_*	W	316
Copper losses in field winding *Р_f_*	W	104
Iron losses *Р_i_*	W	70
Tooth pulsation loss *Р_tp_*	W	2,15
Mechanical losses *Р_m_*	W	40
Additional losses *Р_a_*	W	9,35
Total losses *ΔР*	W	541,5
Input power (not including excitation)	W	1391,5
Efficiency *η*	%	68,5
Moment of inertia *J*	kg.m^2^	0,026
Total mass	kg	70

**Figure 17:**
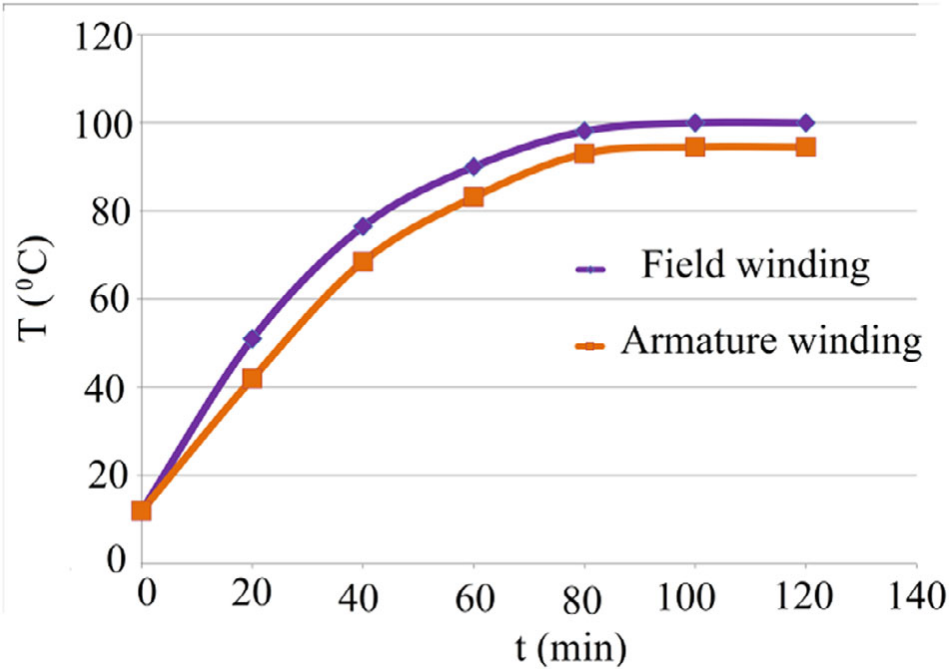
Temperature rise curve.

In addition to the experimental determination of losses and recording the operating characteristics, thermal tests were also conducted. The thermal tests of the DCFR were carried out (**Figure** **17**) under natural cooling conditions, with an ambient temperature of 12 °C and an armature current of Iа = 3.3A. The generator operated in this mode for 100 min and reached a steady‐state temperature of 100 °C, corresponding to insulation class B.

The test results of the DCFR prototype experimentally confirmed the operability of the new design and the effectiveness of the method to reduce the armature reaction field. The originality of this design solution is supported by five Ukrainian invention patents.

The total material costs for manufacturing the DCFR —, i.e., the cost of materials plus the cost of fabricating parts (shaft, poles, end shields, M‐shaped teeth, etc.) — amounted to $1150. Ref. [21] states that “In the European electrical equipment industry, the incidence of material costs on total costs averages 50%.” Accordingly, the total cost of the DCFR generator, including labor, services, etc., comes to $2300. Considering that in serial production the unit cost typically falls by 15–30%, one can estimate the cost of the DCFR generator to be ≈$1610–1946. By comparison, the cost of an induction motor is $1760.62 (*Pn = 1.1 kW, Un = 230/460 V, nn = 850 rpm*) according to data in ref. [22]. Thus, it can be preliminarily concluded that the cost of a DCFR may be roughly equal to that of an induction motor.

### 3.2 Comparing the DCFR with an Analog

According to ref. [23], among all the three‐phase AC motors sold in the EU in 2014 with power ratings from 0.75 kW to 375 kW, the range 0.75–7.5 kW accounted for 62.5% (€3.25 billion). This power range is also critical for DC motors.^[^ ^24^ ^]^

For comparative analysis and to obtain data for further optimization, calculations were performed for the DCFR. The analog used was a DC motor with the following specifications:

*P_n_* = 5.9 kW, *U_n_* = 440 V, *n_n_* = 1490 rpm, *h* = 132 mm, separate excitation with *U* = 190 V, degree of protection IP23, cooling method IC 06, manufactured by Nidec Leroy‐Somer.^[^ ^25^ ^]^

Considering that the DCFR can have two configurations, a comparison was proposed for the following options:

1) A commutator DCFR
2) An electronic controller DCFR

**Figure 18:**
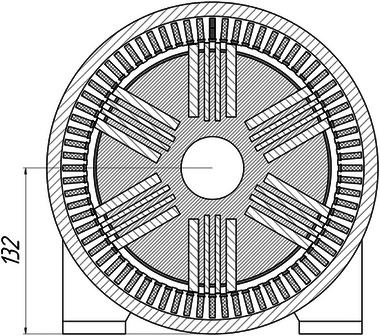
Front view of the commutator DCFR.

**Figure 19:**
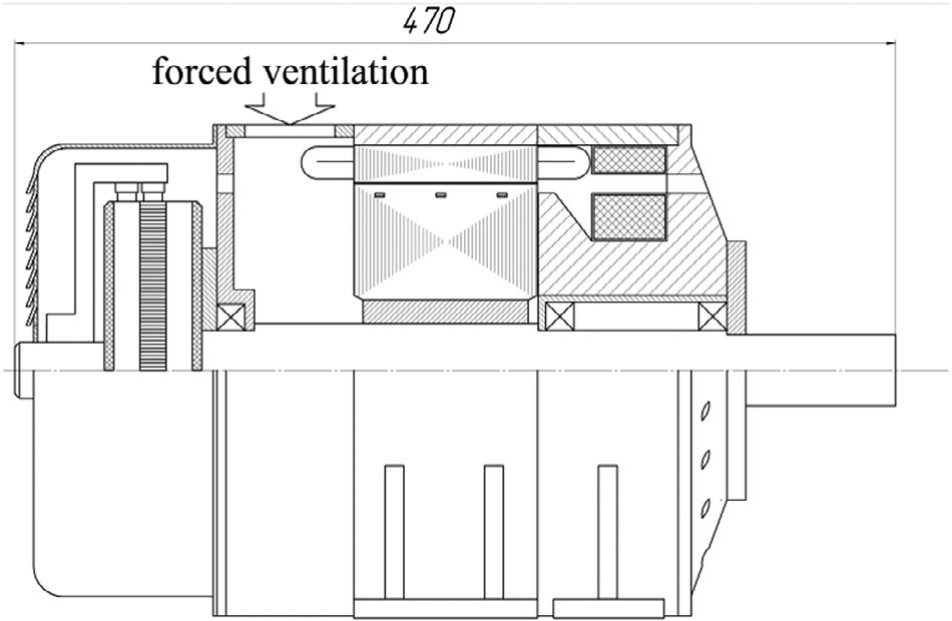
Side view of the commutator DCFR.

The appearance of the commutator DCFR is shown in **Figures** **18** and **19**. In the DCFR design an inverted mechanical commutator is used in which the commutator is stationary and the brush holder with brushes rotates. Preliminary calculations indicated that with an armature diameter of Da = 20 cm, the frame size of the DCFR matches the analog at h = 132 mm. **Table** **3** presents the design parameters and main dimensions of the two DCFR Options. For comparison, the second column shows the recommended ranges of A, Bδ, αδ, 2p and δ in refs. [11, 15] for an armature of Da = 20 cm.

**Table 3:** Design parameters and main dimensions of the DCFR.

Parameter	Unit	From design experience^[^ ^8^, ^13^ ^]^	№1	№2
Rated power *Р*	W		5890	5916
Armature voltage *U*	V		440	440
Rated speed *n*	rpm		1497	1495
Separate excitation type			Separate	
Excitation voltage *U_e_*	V		190	
Armature diameter *D_а_*	cm	20	20	
Commutator diameter *D_c_*	cm		18	–
Axial length of the armature core *ℓ_a_*	cm		9.4	7.9
Linear current density *А*	A cm^−1^	180–290	260	251
Flux density in the air gap *В_δ_*	Т	0.6–0.8	0.72	
Flux density in the armature teeth *В_t_*	Т	1.9–2	1.5	
Flux density in the pole *В_p_*	T	1.45–1.75	1.93	
Flux density in the yoke and flange *В_y_*	T	1.25–1.4	1.7	
Pole arc to pole pitch ratio *α_δ_*		0.62–0.66	0.73	0.87
Back EMF *Е_а_*	V		419	420
Number of poles *2р*		4	12	
Number of parallel paths *2а*		2	2	
Air gap length *δ*	mm	1–1.5	0.4	
Current density of the armature winding ja	A mm^−2^	7–7.2	5.33	5.15
Current density of the excitation winding je	A mm^−2^	4.5–6	3	4
Remagnetization frequency f	Hz	50	149	

The values of the linear current density *A* and the flux density in the air gap *Вδ* were taken as the average of the recommended ranges in ref. [11] to create a baseline calculation for subsequent optimization. The calculation results reveal that the main dimensions have both positive and negative features.

Advantages:

1. an increased pole arc to pole pitch ratio αδ, which reduces the axial length of the armature core ℓa; different αδ values were selected for the two options based on the armature winding commutation conditions;
2. an increased number of pole pairs 2р, which reduces the mass and copper losses in the armature winding;
3. a reduced air gap size, which decreases both the mass and copper losses in the field winding;
4. a reduced windings current density, which minimizes losses in the windings;
5. an increase in the diameter and mass of the commutator does not affect the moment of inertia since the commutator is stationary.

Disadvantages:

1. an increase in the armature diameter Dа also increases the commutator diameter Dc, leading to higher mass and cost;
2. the increased number of poles results in a higher remagnetization frequency f, necessitating a reduction in the flux density in the armature teeth;
3. the segmented poles have greater height, requiring them to be laminated with higher flux density in the poles, which impacts both the mass and the losses in the field winding.

**Table** **4** presents data on losses, efficiency, torque, moment of inertia and commutation.

**Table 4:** Losses, efficiency, torque, moment of inertia, and commutation of the DCFR.

Parameter	Unit	№1	№2
*Current*			
Armature current *I_a_*	А	15.1	14.6
Field current *I_f_*	А	0.66	0.82

*Resistance*			
Armature resistance *R_a Т = 75С_*	Ω	1.3	1.2
Field resistance *R_f Т = 75С_*	Ω	285.3	231.6

*Losses*			
Copper losses in the armature winding *Р_a_*	W	296.4	255.8
Copper losses in the field winding *Р_f_*	W	124.3	155.7
Iron losses *Р_i_*	W	110	91
Brush drop losses *Р_bd_*	W	30	–
Friction losses between the surfaces of the brush to the commutator *Р_fr_*	W	135	–
Tooth pulsation loss *Р_tp_*	W	35	13.6
Mechanical losses (friction & windage) *Р_m_*	W	114	114
Additional losses *Р_a_*	W	34	34
Total losses ΔР	W	878.7	664.1

*Power and efficiency (not taking field excitation into account)*			
Input power to the motor *P_1_ = U·I_a_*	W	6644	6424
Total losses (not taking field excitation into account) *ΔР_‐f_ = ΔР‐ Р_f_*	W	754.4	508.4
Rated power *Р_2_ = Р_1_‐ ΔР_‐f_*	W	5890	5916
Efficiency *ή*	%	88.6	91.8
Torque *М*	N m	37.5	37.7
Moment of inertia *J*	kg m^2^	0.044	0.041

*Commutation*			
Reactive EMF *e_r_*	V	1.05	–
Armature reaction EMF *e_q_*	V	0.63	–
Commutation condition *e_r_ + e_q_*	V	1.69	–
Flux density in the commutation zone *В_q_*	T	0.057	–

Analyzing Tables 3 and 4, it is evident that the increase in the pole arc to pole pitch ratio *α_δ_* in Option №2 allows a reduction in both the axial length of the armature core and the copper losses in the armature winding. The increase in current density in the field winding of Option №2 enables a reduction in the field winding width, its weight, and the magnetic circuit weight (Figure 19). In addition, due to the absence of commutator losses, the total losses in Option №2 are lower than those in Option №1. **Table** **5** presents data on the mass and material costs of the two DCFR options. Prices are given in euros and are based on data from refs. [26, 27]

**Table 5:** Mass of materials and units.

Parameter	Unit	№1	№2
*Mass of copper*			
Armature winding *m_аw_*	kg	4.5	3.9
Field winding *m_fw_*	kg	5.5	4
Commutator *m_c_*	kg	4.6	–
***∑***	kg	14.6	7.9

*Electrical steel mass*			
Stator core *m_sc_*	kg	5.3	4.5
Poles *m_p_*	kg	4.8	4.1
***∑***	kg	10.1	8.6

*Mass of aluminum*			
Yoke (forced ventilation area) *m_yfv_*	kg	1.1	1.1
End shields *m_es_*	kg	2	2
Rotor hub *m_rh_*	kg	3.6	3.1
***∑***	kg	6.7	6.2

*Mass of structural steel*			
Yoke + flange *m_yf_*	kg	25.9	24.9
Shaft *m_s_*	kg	4.5	4.2
***∑***	kg	30.4	29.1

*Mass of units*			
Bearings, brush holder, etc.	kg	3.3	3
Fan motor	kg	3.4	3.4
***∑***	kg	6.7	6.4
Total mass	kg	68.5	59.7

*Cost of materials*			
Copper	€	120	65
Electrical steel	€	9.3	7.8
Aluminum	€	14.5	13.4
Structural steel	€	10	8.7
Total cost (without units)	*€*	153.8	94.9

Note: (*) copper = 8.23 €/kg; electrical steel = 0.916 €/kg; aluminum = 2.16 €/kg; structural steel = 0.33 €/kg.

As can be seen in Tables 4 and 5, eliminating the commutator increases the efficiency and reduces the weight and material cost of the motor in Variant № 2. However, the weight and cost of the electronic controller could negate these advantages. Therefore, a comprehensive economic analysis is required. Due to the limited effects of armature reaction, both DCFR variants can operate with short overload currents equal to 1.5–2. However, while no changes are required for the commutator DCFR to handle such modes, the electronic controller DCFR would require equipment with higher power semiconductor devices, which would increase the cost of the entire DC drive. This also deserves further investigation from both technical and economic perspectives.

**Table** **6** presents a comparison of the DCFR with its DC and AC counterparts.

**Table 6:** Comparison of the DCFR with DC and AC analogs.

Parameter	Unit	DC	AC (IE2)	DCFR	
Manufacturer		Leroy‐Somer	ABB^[^ ^28^ ^]^		
Model		MS1322S	M2BAX 132SA	№1	№2
Frame size *h*	mm	132	132	132	132
Rated power *Р_n_*	kW	5.9	5.5	5.9	5.9
Rated speed *n_n_*	rpm	1490	1457	1497	1495
Rated torque *М_n_*	N.m	38	36	37	38
Rated current *I_n_*	А	16	11.7	15	15
Moment of inertia *J*	kg.m^2^	0.04	0.026	0.04	0.04
Total weight *m*	kg	76	59	69	60
Efficiency η (not including excitation)	%	85	87.7	88.6	91.8

As Table 6 shows, the DCFR design has lower weight and higher efficiency compared to the brush DC motor. Compared to the induction motor, Variant No. 1 is 15% heavier, while Variant No. 2 is only 2% heavier. At the same time, both DCFR variants have greater efficiency than the AC counterpart. It is also worth noting that the data presented are for non‐optimized DCFR designs. These designs have significant potential ranges of electromagnetic loads, current density, commutation conditions and temperature. This provides extensive opportunities for optimization to meet specific requirements.

### 3.3 Magnetic Field and Stationary Temperature Field Analyses of the DCFR

**Figure 20:**
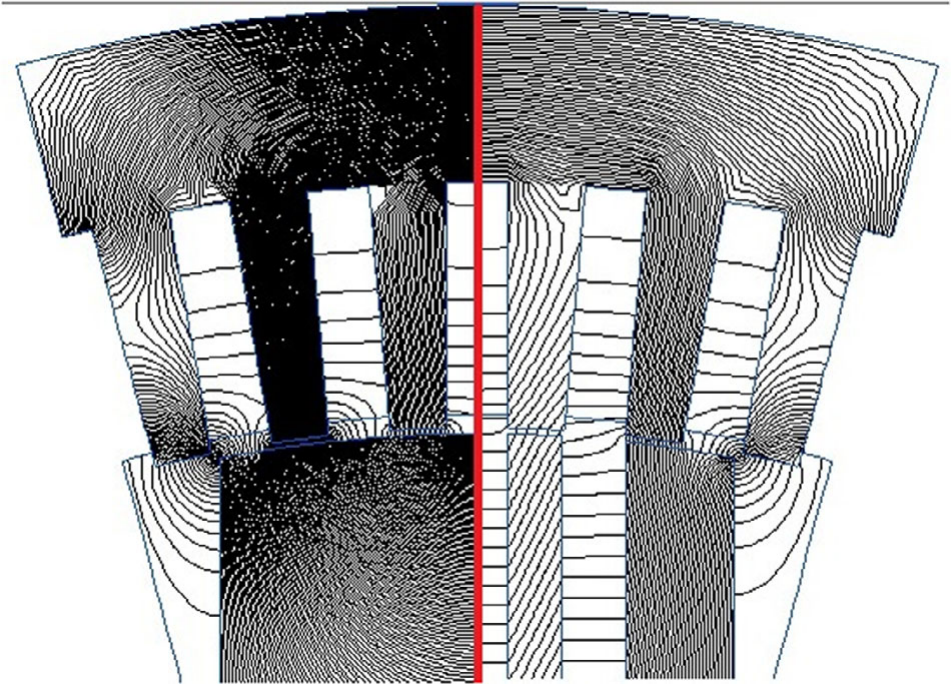
Distribution of magnetic lines of force due to only armature MMF in non‐segmented (left side) and segmented (right half) DCFR poles.

**Figure 21:**
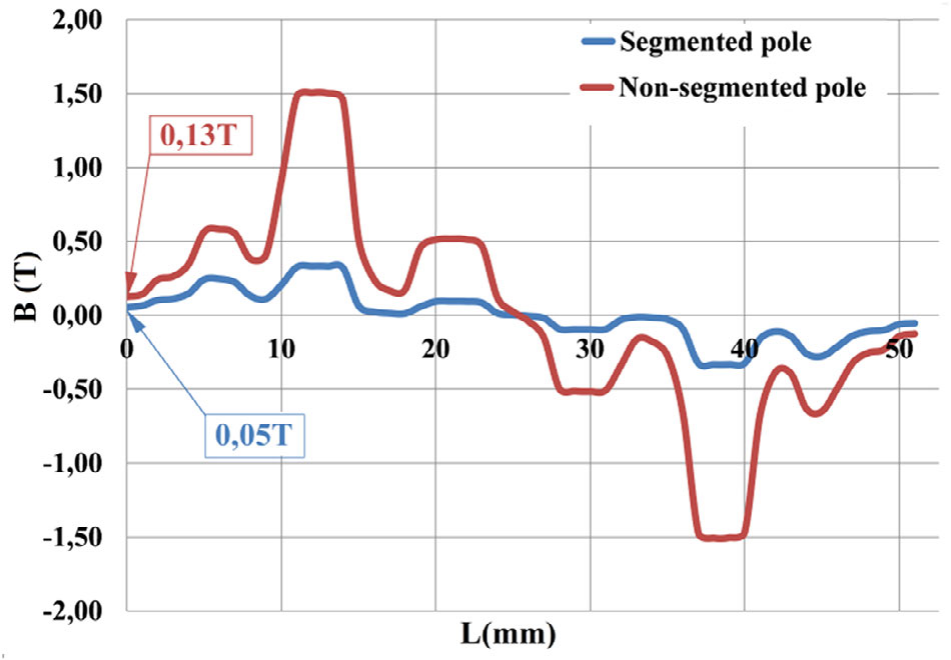
Air‐gap flux density due to armature reaction for non‐segmented and segmented poles.

Given that the electromagnetic loads for Options № 1 and № 2 are similar, this section focuses only on commutator DCFR Option №1. We analyzed the magnetic field and temperature field using the finite element method in the QuickField software package. **Figure** **20** shows the distribution of magnetic lines of force due to only armature MMF, and compares the non‐segmented (left side) and segmented (right side) DCFR poles. For the same case, **Figure** **21** illustrates the air‐gap flux density due to armature reaction for non‐segmented and segmented poles.

**Figure 22:**
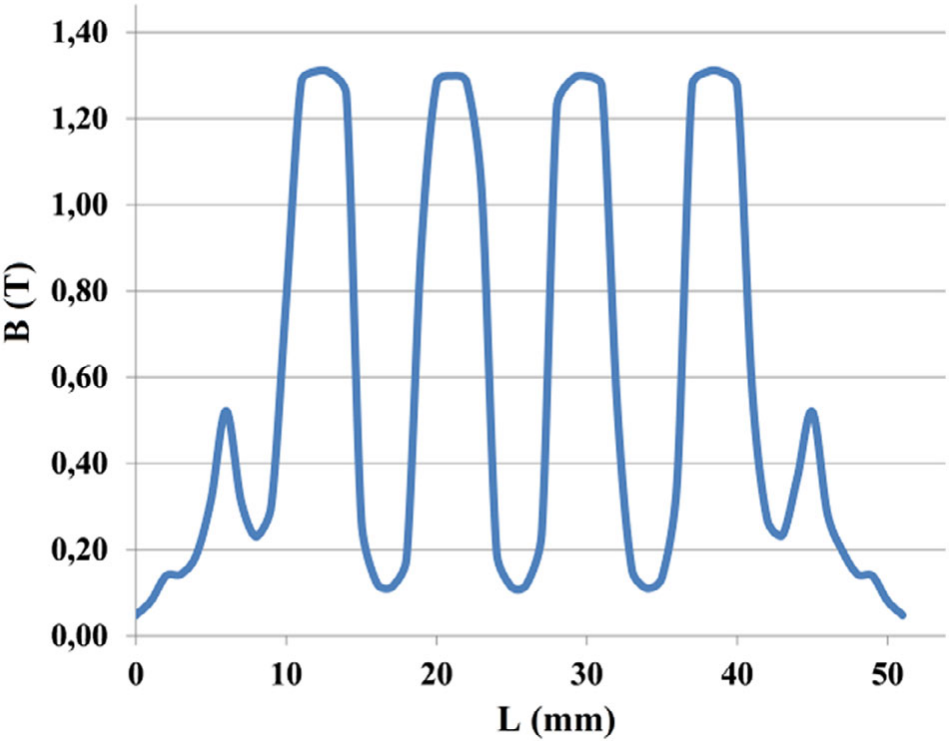
Airgap flux density distribution in the no‐load condition (only the field winding is excited).

**Figure 23:**
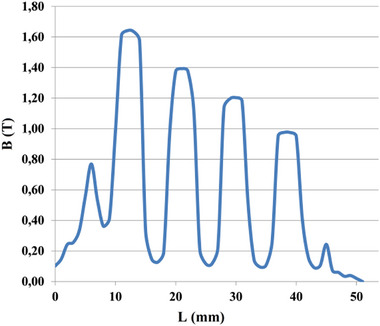
Resultant airgap flux density distribution in the load condition.

The figures show that pole segmentation significantly reduces the armature reaction flux. The flux density in the commutation zone from the action of the armature reaction is *B_q_* = 0.05 T, which closely aligns with the calculated value of *B_q_* = 0.057 T (Table 4). Furthermore, a comparison of the Bq values for segmented and non‐segmented options indicates that the magnetic flux density in the non‐segmented option is 2.6 times higher. **Figures** **22** and **23** present the distribution of the air‐gap flux density in the no‐load and load conditions.

The data to calculate the stationary temperature field of the DCFR in the nominal operating mode are presented in **Table** **7**.

**Table 7:** Data for the stationary temperature field calculation.

Parameter	Unit	Value
Heat transfer coefficient *h*	W m^−^^2^ K	60
Ambient temperature *Т_о_*	C	40

Power volume of the heat source		
Armature winding *Q_a_*	W m^−3^	211 350
Upper field winding *Q_f1_*	W m^−3^	50 171
Lower field winding *Q_f2_*	W m^−3^	91 016

Insulation class: F, with 155 °C maximum temperature.

**Figure 24:**
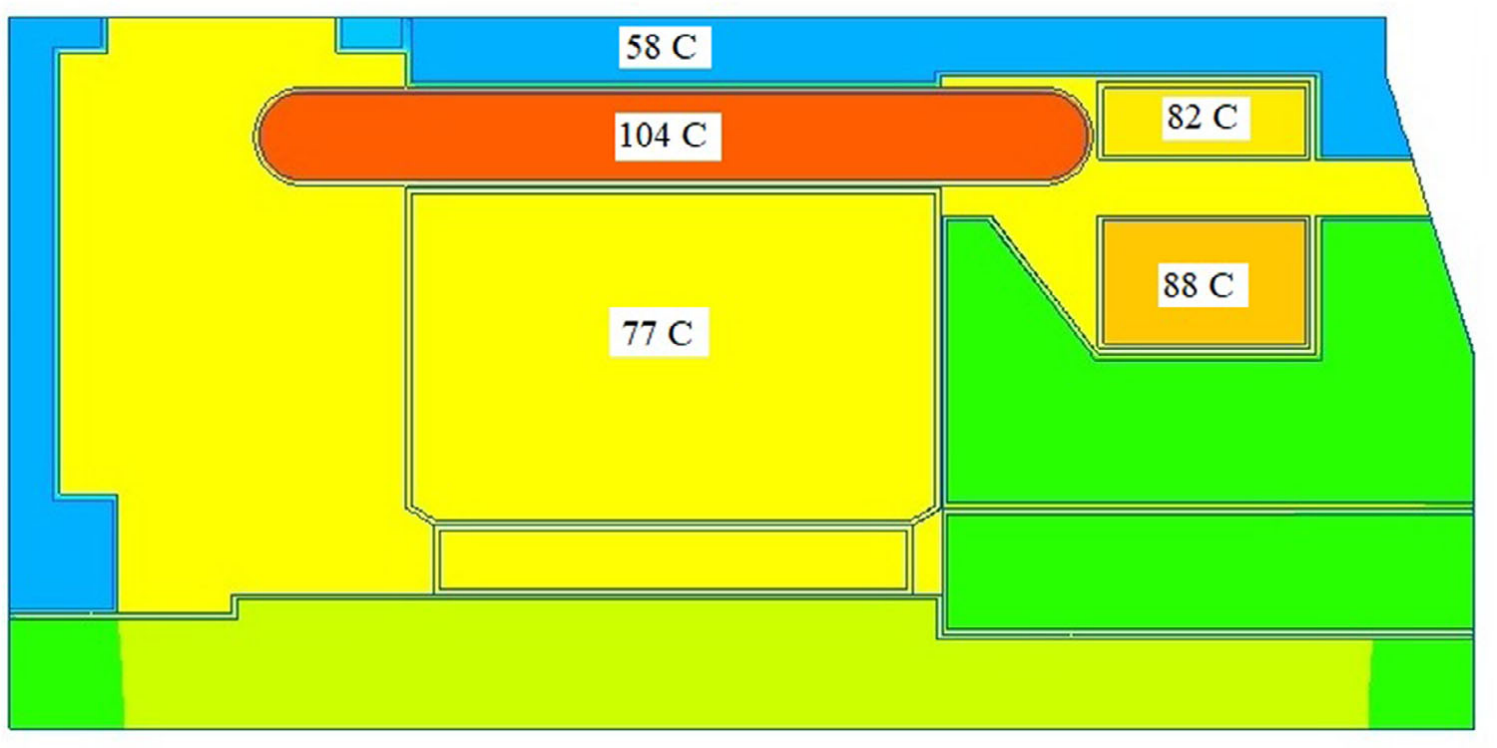
Temperature distribution in the DCFR in the nominal operating mode.

The results of the stationary temperature field calculation are shown in **Figure** **24**.

Thermal analysis of the motor with a 1.5 overload reveals the following temperature values:

1. Armature winding *T_a_* = 133 °C
2. Upper field winding *T_f1_* = 97 °C
3. Lower field winding *T_f2_* = 104 °C

The analog manufacturer's catalog states ″Motors can tolerate an overload between 0 and the rated speed of:

1.  1.6 times the rated torque for ≈20 s every 5 min, or
2.  1.6 times the rated torque for 1 min, twice an hour.″

Based on the data obtained, it can be assumed that the DCFR could operate under these conditions for a longer period. This is supported by both the thermal calculation data and the armature winding commutation condition data. This potential capability could result in significant economic benefits.

## 4 Results and Discussion

### 4.1 Manufacturing DCFR Technology

Eliminating compensating windings and commutating poles significantly simplifies the design of the DCFR, which is divided in three independent units:

  1. A yoke with a stator core and an armature winding (Figure 1, I),
  2. A flange with a field winding (Figure 1, II),
  3. A shaft with a rotor hub and poles (Figure 1, III).

Each unit is manufactured independently and they are only assembled in the final assembly stage. This approach enables the production of DCFRs to be almost entirely automated and minimizes manual labor.

### 4.2 Analysis of the Results

An analysis of the research materials presented above indicates that the DCFR has a mass, efficiency and cost comparable to those of induction motors (see Table 6). As was noted in Section 2.1, a DCFR can be equipped with either a commutator or an electronic controller. Both configurations have been tested and confirmed to be functional.

However, the electronic controller configuration has a significant drawback: a large number of power semiconductor switches, which can substantially increase its price compared to the cost of a variable‐frequency drive. Solving this problem requires a comprehensive approach that considers the cost of both the motor and the electronic controller, but it is not addressed in this article.

As a result, this study treats the commutator option as the primary one. Judging from the publications available (see Section 2.7), there is a longstanding negative perception of commutators in the scientific community. In general‐purpose motors, the commutator is viewed as a single entity along with commutating poles and a compensating winding. Removing these three components in BLDC motors reduces their size and increases their efficiency, but it does not make them cheaper.

A rough impression of the relative prices can be gained by comparing a range of 100 W, 3000 rpm DC and AC micromachines (**Table** **8**). A power rating of 100 W was chosen because brush DC motors with this power do not use commutating poles.

**Table 8:** Comparison of prices for DC and AC micromotors.

Parameter	AC Single‐phase	Brushed DC shunt wound	Brushed DC with permanent magnets	BLDC+ Drive
Manufacturer	Oriental motor	Parvalux	Crouzet	Crouzet
Part No	5IK90A‐DW3E	111295SH	89890011	80180050
***Cost ($)***	***167***	***243***	***301***	***634***

The difference between the DCFR and the BLDC lies in the fact that the DCFR retains the commutator, which is an excellent electromechanical converter. As confirmation, one can cite the conclusion of a study by ABB Corporation^[^ ^29^ ^]^ that “today, depending on the application involved, the useful lifetime of brushes in DC motors is ≈7000 to 12 000 h, thanks to the sophisticated collectors, carbon brushes and optimized field supply units used. Depending on the mechanical conditions involved, the relubrication intervals for the bearings of DC/AC motors may be shorter than the useful lifetime of the brushes in DC motors.”

Moreover, it is precisely the commutator version of the DCFR that may be in demand for solar pumps (see the Introduction). In ref. [7], one of the reasons for customer distrust of BLDC explicitly regards solar pumps: “In addition, DC‐type pumps have had a lower rate of acceptance than AC pumps due to a number of instances of faulty wiring, electronic circuit failure, unsafe DC voltages and terminals that did not offer a solid electrical connection.”

If necessary, a farmer can independently rewind the winding of an induction motor by looking up the winding diagram on the internet, and in the same way can rewind the winding and handle the commutator soldering in a DCFR. For these operations, it is not necessary to have skills in repairing complex electronic equipment. Given the comparable price, the savings on an inverter and solar PV modules could be a strong argument in favor of the DCFR. Of course, an MPPT solar controller is still required, but its cost is significantly lower than that of an inverter.

In essence, the DCFR remains a brush DC motor with its own strengths and weaknesses. Like any brush DC motor, the DCFR has an upper rotational speed limit of ≈4000 rpm. According to ref. [30], “the wear of brush at 5000 rpm increases to about five times larger than at 1000 rpm.” A strong point of the DCFR is its effectiveness in projects that require direct‐drive high‐torque low‐speed motors. As is known, a highly efficient low‐speed motor must have a large number of poles. Unlike multipole synchronous motors with permanent magnets, a commutator DCFR with the same number of poles does not require permanent magnets. Therefore, in addition to being a general‐purpose motor with a rotational speed of 1000–3000 rpm, the DCFR can also be used in cranes, elevators and machine‐tool drives, and for transferring rotation in a sealed volume through a non‐magnetic partition (a type of “magnetic drive pump” but without permanent magnets).

In addition, one should take into account the significant potential of the linear DCFR design for maglev electric transport, rotary tables, multi‐axis manipulators and more. The use of a DCFR as a generator in wind turbines appears highly promising, especially in megawatt‐class wind generators.

Maintenance of a DCFR motor does not differ from that of a classic brush DC motor. However, the maintenance and overall repair time is reduced by the absence of commutating poles and compensating windings in the DCFR design. The service life of the DCFR and its limitations under various environmental conditions are the same as those of a classic brush DC motor.

## Conflict of Interest

The authors declare no conflict of interest.

## Data Availability

The data that support the findings of this study are available from the corresponding author upon reasonable request.
